# TRPV4 affects visual signals in photoreceptors and rod bipolar cells

**DOI:** 10.3389/fncel.2024.1404929

**Published:** 2024-06-05

**Authors:** Ye Long, Maxim Kozhemyakin, Samuel M. Wu, Ji-Jie Pang

**Affiliations:** Department of Ophthalmology, Baylor College of Medicine, Houston, TX, United States

**Keywords:** TRPV4, rod bipolar cell, photoreceptor, light response, ERG, patch-clamp, immunocytochemistry, confocal microscopy

## Abstract

**Introduction:**

Mechanical sensitive channels expressed in mammalian retinas are effectors of elevated pressure stresses, but it is unclear how their activation affects visual function in pressure-related retinal disorders.

**Methods:**

This study investigated the role of the transient potential channel vanilloid TRPV4 in photoreceptors and rod bipolar cells (RBCs) with immunohistochemistry, confocal microscopy, electroretinography (ERG), and patch-clamp techniques.

**Results:**

TRPV4 immunoreactivity (IR) was found in the outer segments of photoreceptors, dendrites and somas of PKCα-positive RBCs and other BCs, plexiform layers, and retinal ganglion cells (RGCs) in wild-type mice. TRPV4-IR was largely diminished in the retinas of homozygous TRPV4 transgenic mice. Genetically suppressing TRPV4 expression moderately but significantly enhanced the amplitude of ERG a- and b-waves evoked by scotopic and mesopic lights (0.55 to 200 Rh*rod^−1^ s^−1^) and photopic lights (10^5^–10^6^ Rh*rod^−1^ s^−1^) compared to wild-type mice in fully dark-adapted conditions. The implicit time evoked by dim lights (0.55 to 200 Rh*rod^−1^ s^−1^) was significantly decreased for b-waves and elongated for a-waves in the transgenic mice. ERG b-wave evoked by dim lights is primarily mediated by RBCs, and under voltage-clamp conditions, the latency of the light-evoked cation current in RBCs of the transgenic mice was significantly shorter compared to wild-type mice. About 10% of the transgenic mice had one eye undeveloped, and the percentage was significantly higher than in wild-type mice.

**Conclusions:**

The data indicates that TRPV4 involves ocular development and is expressed and active in outer retinal neurons, and interventions of TRPV4 can variably affect visual signals in rods, cones, RBCs, and cone ON BCs.

## Introduction

A variety of retinal disorders are associated with the elevation of intraocular pressure (IOP) and changes in the external pressure, such as glaucoma, traumatic retinal injury (TRI), and visual impairments occur during air travel, diving, and mountain hiking (reviewed by Jonas et al. (2017), Allison et al. (2020), Evans et al. (2021), and Pang (2021)). The retina has been reported to express various transient receptor potential channels (TRPs) (reviewed by Yang et al., 2022; Krizaj et al., 2023), and some TRPs are also known as mechano-gated channels that may be directly activated by membrane tension (Liu and Montell, 2015), however, the role of mechanical sensitive channels (MSCs) in visual functions and pressure-related retinal diseases has been unclear.

Transient receptor potential channel vanilloid TRPV is a subset of TRPs, including six members TRPV1-6. TRPVs have a permeability (P) to Ca^2+^ higher than PNa^+^, and the PCa: PNa for TRPV4 is 6–10. TRPV4 may be activated by mechanical and osmotic pressure, touch, warm temperature, and other factors, and it mediates cation currents that reverse at ~0 mV (Strotmann et al., 2000; Suzuki et al., 2003b; Nilius et al., 2004; O'Neil and Heller, 2005; Cao et al., 2009; Gao et al., 2019; Kashio and Tominaga, 2022). These properties allow TRPVs to mediate membrane depolarization and Ca^2+^-related physiological and pathological activities. RGCs in the peripheral and paracentral retina are the most vulnerable to pressure stresses (Jonas et al., 2017; Allison et al., 2020; Evans et al., 2021; Pang, 2021), and TRPV4 has been found in the retinal ganglion cell layer (GCL) and plexiform layers of the mouse (Ryskamp et al., 2011; Sappington et al., 2015), porcine (Taylor et al., 2016), and monkey retina (Gao et al., 2019). The optic nerve head exhibited mRNAs of TRPV4 (Choi et al., 2015). The level of TRPV mRNAs in isolated RGCs of 7–15 μm in diameter from the mouse retina was TRPV4 > TRPV2 > TRPV3 and TRPV1 (Lakk et al., 2018). RGCs in the mouse (Ryskamp et al., 2011) and primate retina (Gao et al., 2019) can be activated by micromolar TRPV4 agonists GSK1016790A and 4α-phorbol 12,13-didecanoate (4αPDD), exhibiting membrane depolarization and higher firing rate. In cultured RGCs, TRPV4 agonists evoked calcium influxes and were associated with apoptosis of the neurons (Ryskamp et al., 2011). TRPV4 antagonist RN1734 has been tested in retinal slices in culture and revealed a neuroprotective role in the porcine retina (Taylor et al., 2016). These observations have confirmed the expression and potential neurodegenerative role of TRPV4 in RGCs.

On the other hand, the elevation of IOP in rat and mouse glaucoma models also damages ribbon synapses of photoreceptors and BCs (Cuenca et al., 2010; Fuchs et al., 2012; Park et al., 2014) and dendrites of BCs and horizontal cells (Noailles et al., 2022), and the dysfunction of rod bipolar cells (RBCs) (Shen et al., 2019) and rod signals in AII amacrine cells (Pang et al., 2015) occur before the loss of RGCs. Recent data have identified some pressure-evoked cation currents in vertebrate photoreceptors and mammalian BCs (20;27) that reverse at ~0 mV. Some TRPV4 immunoreactivities were found in the processes and somatic membrane of the photoreceptors and RBCs. To better understand the role of TRPV4 in the outer retina, in this study, we explored the expression of TRPV4 in wild-type and TRPV4 knockout mice with immunocytochemistry and confocal microscopy and investigated the functions of TRPV4 in photoreceptors and ON BCs with patch-clamp recording and electroretinography (ERG) in fully dark-adapted conditions.

## Methods

### Animals and preparations

All procedures were carried out in strict accordance with the recommendations in the Guide for the Care and Use of Laboratory Animals of the National Institutes of Health, ARVO Statement for the Use of Animals in Ophthalmic and Vision Research, and related regulations of Institutional Animal Care and Use Committee. Animals were 3-7-month old mice, males and females, including C57BL/6 J (wide-type mice) purchased from Jackson Laboratory (Bar Harbor, ME) and TRPV4 transgenic mice (C57BL/6 N-Trpv4^em1(cre/ERT2)Amc^/J, stock# 029582, Jackson Laboratory) (Suzuki et al., 2003a,b) maintained in our animal facility. The homozygotes (namely *TRPV4^−/−^*) exhibited a reduced level of TRPV4 expression (see results). Chemicals were purchased primarily from Sigma-Aldrich (St. Louis, MO) and Tocris Bioscience (Bristol, United Kingdom) except otherwise specified.

### Patch-clamp recording of bipolar cells

All procedures were performed under infrared (~1 mm) illumination with dual-unit Nitemare (BE Meyers, Redmond, WA) infrared scopes. The whole-cell patch-clamp recording (Pang et al., 2010a, 2012), preparation of living retinal slices (Werblin, 1978; Wu, 1987), light simulation, immunofluorescence, and confocal microscopy (Pang et al., 2018; Gao et al., 2019) essentially followed procedures described in previous publications.

Animals were dark-adapted for 1–2 h before the related experiment. The Ames medium in the recording chamber was oxygenated and maintained at 34°C with a temperature control unit (TC 324B, Warner Instruments, CT). The controller was wired with DigiData1322A to record and monitor the temperature. Axopatch 700A and 700B amplifiers were connected to DigiData 1322A interfaces and operated by the pClamp software v9.2 and v10.3 (Axon Instruments, Foster City, CA). Patch pipettes had 9–12 MΩ tip resistance when filled with an internal solution containing 112 mM Cs-methanesulfonate, 12 mM CsCl, 5 mM EGTA, 0.5 mM CaCl_2_, 4 mM ATP, 0.3 mM GTP, 10 mM Tris, and 0.5% Lucifer yellow, adjusted to pH 7.3 with CsOH. For current-clamp and some voltage-clamp recordings, the pipettes were filled with internal solutions containing: 112 mM K-gluconate, 10 mM KCl, 10 mM EGTA, 10 mM HEPES, 0.5 mM CaCl_2_, 1 mM MgCl_2_, 4 mM Na_2_-ATP, 0.3 mM Na_3_-GTP, and 0.5% Lucifer yellow, adjusted to pH 7.3 by KOH. The internal solution and external normal Ringer’s solution yield a chloride reversal potential (E_Cl_) of −59 mV at room temperature. Recorded cells were visualized by Lucifer yellow fluorescence with a confocal microscope (LSM 510 and LSM 800, Carl Zeiss, Germany).

A photostimulator delivered light spots of a diameter of 600–1,200 μm and 500 nm wavelength (λ_max_ = 500 nm, full width-half max 10 nm) at a series of intensities (−10 to −1 log I) to stimulate the retina via the epi-illuminator of the microscope (Maple and Wu, 1998; Pang et al., 2002, 2010b). Since we delivered uncollimated light beams through an objective lens of a large numerical aperture (Zeiss 40x/0.75 water), the incident light could enter the retina in many directions and, thus, had a minor photoreceptor self-screening effect (Field and Rieke, 2002). The intensity of unattenuated (0 in log unit (log I)) 500 nm light from a halogen light source was 4.4 × 10^5^ photons.μm^−2^.sec^−1^. The light intensity was transformed into the unit of photoisomerization per rod per second (Rh*rod^−1^ s ^−1^) with a rod cross-section of 0.5 μm^−2^ (Howes et al., 2002) and a rod integration time of 0.4 s (Baylor, 1987).

### Electroretinography (ERG)

ERG recording followed previously established protocols (Pennesi et al., 2003; Abd-El-Barr et al., 2009; Tse et al., 2015). The mouse was dark-adapted overnight, anesthetized, and kept on a warm pad of 30–42°C. Under dim red-light illumination, we applied a single drop of 1% tropicamide and 2.5% phenylephrine to dilate the pupils and a drop of 0.5% proparacaine hydrochloride for corneal anesthesia. Then, we placed the mouse with the warm pad into a Ganzfeld dome coated with highly reflective white paint (Munsell Paint, New Windsor, NY, United States) on the inner surface. A small amount of 2.5% methylcellulose gel was applied to the eye to ensure the contact of a platinum recording electrode with the center of the cornea. Two similar platinum electrodes were placed in the forehead and tail as the reference and ground electrodes, respectively. The mouse was kept in complete darkness for 5 min before testing. ERG signals were amplified with a Grass P122 amplifier (bandpass 0.1–1 kHz; Grass Instruments, West Warwick, RI, United States). Data were digitized with a computer data acquisition unit (USB-6216, National Instruments, TX) at a sampling rate of 10 kHz, and trials were averaged and analyzed with custom Matlab code (Mathworks, Natick, MA, United States). Flashes for scotopic measurements were generated by cyan light emitting diodes of 503 nm peak wavelength, calibrated with a photometer (ILT1700 International Light, MA), and converted into Rh*rod^−1^ s ^−1^ by 1 scot cd m^2^ = 581 Rh*rod^−1^ s ^−1^ (Tse et al., 2015). A series of metal plates with holes of varying diameters and glass neutral density filters were used to attenuate the light intensity. As the light intensity increased, the number of trials was reduced, and the interval between flashes was increased. Each recording was averaged from 20 to 40 trials for light intensities of 0.055 to 0.025 Rh*rod^−1^ s ^−1^ with an interval of 2 s, 2–5 trials for lights of 0.6 to 200 Rh*rod^−1^ s ^−1^ with an interval of 5–30 s, and one trial for photopic lights of 10^4.81^ and 10^6.17^ Rh*rod^−1^ s ^−1^ with an interval of 45 s and 105 s, respectively. The light duration for dim lights was 0.5 - 5 ms. Photopic stimuli were white lights generated by 1,500-W Novatron (Dallas, TX) xenon flash lamps with a duration of 5 ms.

### Immunocytochemistry and retrograde labeling of RGCs

Double- and triple-immuno-labeling followed our published experimental protocols (Zhang et al., 2005; Pang et al., 2010a,b, 2012; Pang and Wu, 2011; Gao et al., 2019). We fixed the retinas (~30) with 4% paraformaldehyde in phosphate buffer (pH 7.4) for 1–2 h at room temperature or 4°C overnight and then blocked them with 10% donkey serum (Jackson ImmunoResearch, West Grove, PA) in TBS (D-PBS) with 0.5% Triton X-100 (Sigma-Aldrich) and 0.1% NaN3 (Sigma-Aldrich) for 2 h at room temperature or 4°C overnight to reduce nonspecific labeling. Then, we embedded the retina in low gel-point agarose (Sigma-Aldrich), trimmed it into a 10 × 10 × 10 mm^3^ block, glued the block onto a specimen chamber mounted on a vibratome (Pelco 102, 1,000 Plus; Ted Pella, Inc., Redding, CA), and subsequently cut it into 40-μm-thick vertical sections in PBS solution (Pang and Wu, 2011). For staining, retinal tissues were incubated in primary antibodies in the presence of 3% donkey serum-TBS for 3 to 5 days at 4°C. After several rinses, we transferred them into Cy3-, Cy5-, or Alexa Fluor 488-conjugated streptavidin (1:200, Jackson ImmunoResearch), with Cy3- and/or Cy5-conjugated secondary antibodies (1:200, Jackson ImmunoResearch) and/or Alexa Fluor 488-conjugated secondary antibodies (1:200, Molecular Probes, Eugene, OR), in 3% normal donkey serum-TBS solution at 4°C overnight. A nuclear dye, TO-PRO-3 (0.5 μL/mL, Molecular Probes, Eugene, Oregon), was used with the secondary antibody to visualize the nuclei of cells. After extensive rinsing, retinal preparations were cover-slipped. Two small pieces of filter paper (180-μm thick, MF-membrane filters, Millipore, Billerica, MA) were mounted beside flat-mount retinas to prevent them from being over-flattened. Control tests were also executed without using the primary antibody or with the wrong primary antibodies to confirm the results, and secondary antibodies did not generate specific signals in retinal layers.

RGCs were identified with a retrograde labeling technique previously established by Pang and colleagues (Pang et al., 2010b; Pang and Wu, 2011). Briefly, eyeballs of dark-adapted animals were enucleated under the illumination of dim red light. The nerve stump of the freshly dissected eyeball was dipped into a small drop (3 μL) of 3% Lucifer yellow (Sigma) and/or 8% neurobiotin (NB, Vector Laboratories, CA) in the internal solution (Pang et al., 2010b) for 20 min. Then, the eyeball was thoroughly rinsed with the oxygenated Ames medium (Sigma) to remove the extra dye and dissected under infrared illumination. The dark-adapted eyecup with intact retina and sclera tissue was transferred into fresh oxygenated Ames medium and kept at room temperature for 40 min under a 10 min-dark/10 min-light cycle. Subsequently, the whole retina was isolated from the sclera, fixed in darkness for 30–45 min at room temperature, and visualized with Cy3-, Cy5-, or Alexa Fluor 488-conjugated streptavidin (1:200, Jackson ImmunoResearch). The technique brightly labeled the entire population of RGCs in the mouse retina (Pang et al., 2010b; Pang and Wu, 2011).

### Antibodies

Rabbit anti-TRPV4 antibodies (LS-C135, 1: 200; LS-A8583 1:200 and LS-C94498 1: 100) (Ryskamp et al., 2011; Gao et al., 2019) were purchased from LifeSpan Biosciences, Inc. (Seatle, WA). LS-C94498 was raised against a synthetic peptide from the cytoplasmic domain (aa100-150) of mouse TRPV4 conjugated to an immunogenic carrier protein. LS-A8583 targets a synthetic 20-amino acid peptide from the internal region of human TRPV4, and LS-C135 was raised against rat TRPV4 (Q9ERZ8, aa853-871, peptide immunogen sequence: CDGHQQGYAPKWRAEDAPL). The specificity of LS-A8583 and LS-C94498 for labeling retinal TRPV4 was confirmed in TRPV4 knockout mice in a previous report (Ryskamp et al., 2011), and the specificity of LS-C135 was demonstrated in this work (see results). LS-C135 antibody provided the best signal-to-noise ratio in the primate retina (Gao et al., 2019) and was primarily used in this study.

Protein Kinase-C alpha (PKCα) is a classic marker of RBCs (Pang et al., 2013), and we used two PKCα antibodies. The polyclonal anti-PKCα antibody was purchased from Sigma (P4334, 1: 1000, rabbit), which was tested in immunoblotting in rat brain extract, and it recognized a heavy band at ~76 kDa and a very weak band at 40 kDa. The staining was specifically inhibited by PKCα immunizing peptide (659–672). The monoclonal anti-PKCα antibody from BD transduction (610,107, Clone 3/PKCα (RUO), 1: 200, mouse) identified a single band at 82 kDa from a rat cerebrum lysate close to the predicted molecular weight of PKCα 76–93 kDa. The specificity of these primary antibodies has been demonstrated in previous studies, and their staining patterns in our results were like those reports. Controls were also processed with blocking peptides or without primary antibodies. All controls did not show positive results.

### Statistical analysis

Data were analyzed by Sigmaplot (v12 and v15, Systat, Point Richmond, CA), Clampfit (v10.3 and v9.2, Axon Instruments, Foster City, CA), Matlab, and Microsoft Excel and presented as *mean ± s.d*. Two-tail Student *t-test* was used for analyzing statistical significance between paired data groups. The α level to reject the null hypothesis was 0.05.

## Results

### TRPV4 expression in the outer retina

**Figure 1:**
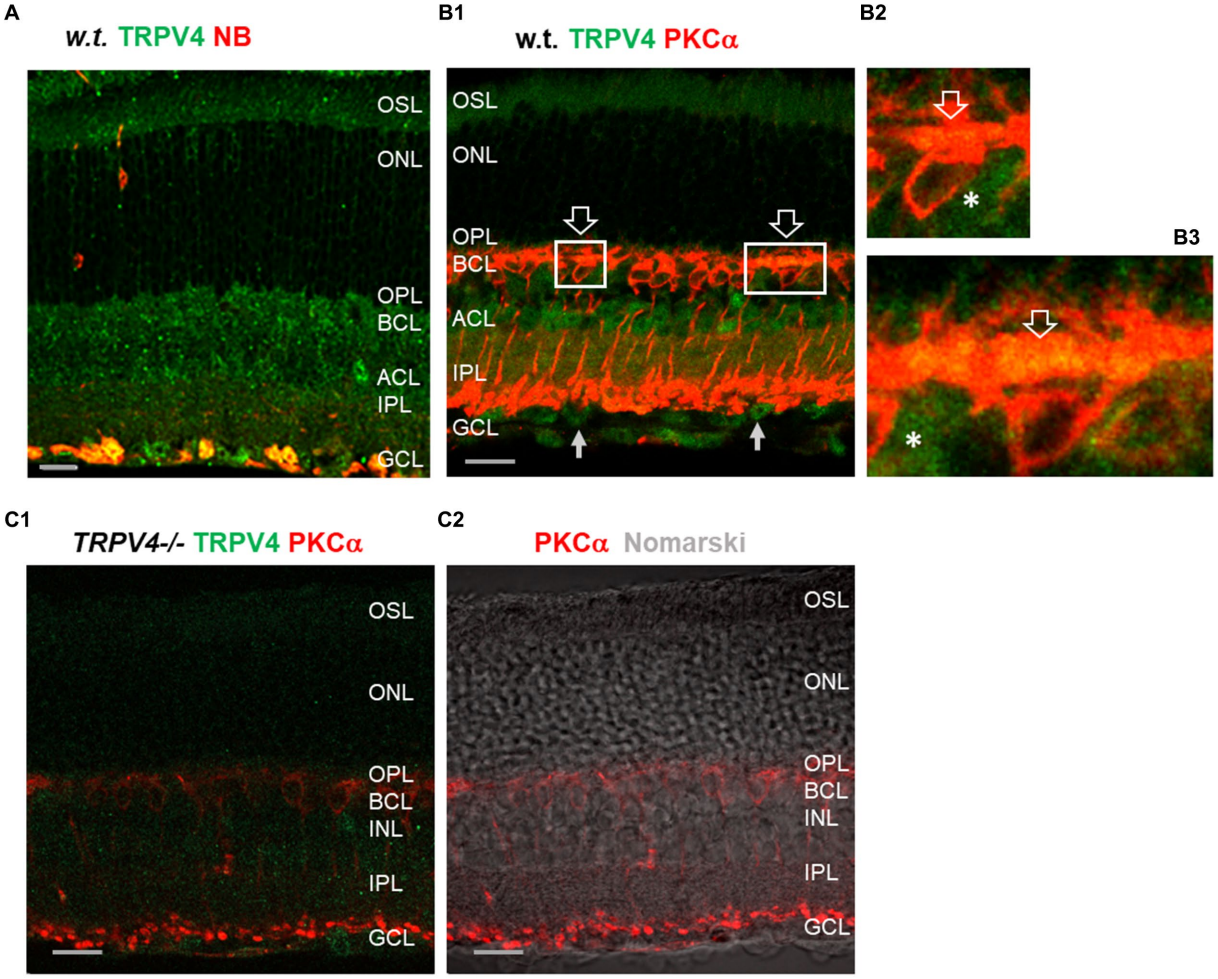
TRPV4 expression in wild-type (*w.t.)* and *TRPV4^−/−^* mice. The retinal slices were labeled for TRPV4 (green) and PKCα, and retinal ganglion cells (RGCs) were retrogradely labeled with neurobiotin (NB, red, **A**). **(A,B)** In wild-type (*w.t.*) mice, TRPV4 immunoreactivity is consistently present in the outer segment layer (OSL, **B1**), outer plexiform layer (OPL), bipolar cell layer (BCL), inner nuclear layer (INL), amacrine cell layer (ACL), inner plexiform layer (IPL), and retrograde-labeled RGCs (yellow, **A**) in the ganglion cell layer (GCL). Some TRPV4 immunoreactivity colocalizes with PKCα in dendrites and somatic membrane of rod bipolar cells in the OPL (**B1–B3**, open arrow), somas of BCs negative to PKCα in the BCL (asterisks, **B2,B3**), and somas in the INL and GCL (arrow, **B1**). **(B2,B3)**: Insets of **B1**. **(C)** In *TRPV4^−/−^* mice, TRPV4 signals are absent in the OSL and ONL and largely diminished in other layers. The scale bars are 20 μm.

We labeled retinas from more than 20 mice with antibodies against TRPV4 and PKCα (Figure 1) and retrograde tracer neurobiotin (NB) for the identification of retinal ganglion cells (RGCs). In wild-type mice (Figures 1A,B), TRPV4 was expressed weakly in the outer segment layer (OSL) of photoreceptors and brightly in the outer and inner plexiform layers (OPL and IPL, respectively), inner nuclear layer (INL), and retrogradely identified RGCs (Figure 1A) in the ganglion cell layer (GCL). In retinal slices double labeled for TRPV4 and PKCα, a marker for rod bipolar cells (RBCs), TRPV4 signals were present in the dendrites and somatic membrane of RBCs and somas of some other BCs (Figure 1B). In *TRPV4^−/−^* mice (Figure 1C), TRPV4 immunoreactivity was absent in the OSL and largely diminished in OPL, IPL, INL, and GCL, demonstrating the specificity of the antibody and different expression levels of TRPV4 in retinal layers. The data indicates the expression of TRPV4 in photoreceptors, BCs, and RGCs.

Besides, ~10% of *TRPV4^−/−^* mice (3/34 mice) had one eye undeveloped. The eyelids were recognizable, but the eyeball was absent. The animals did not show other defects at the macroscopic level. In more than 100 wild-type mice, we did not find similar pathology (0%) (*p* = 0.003).

### TRPV4 affected the amplitude and implicit time of ERG a- and b-wave

**Figure 2:**
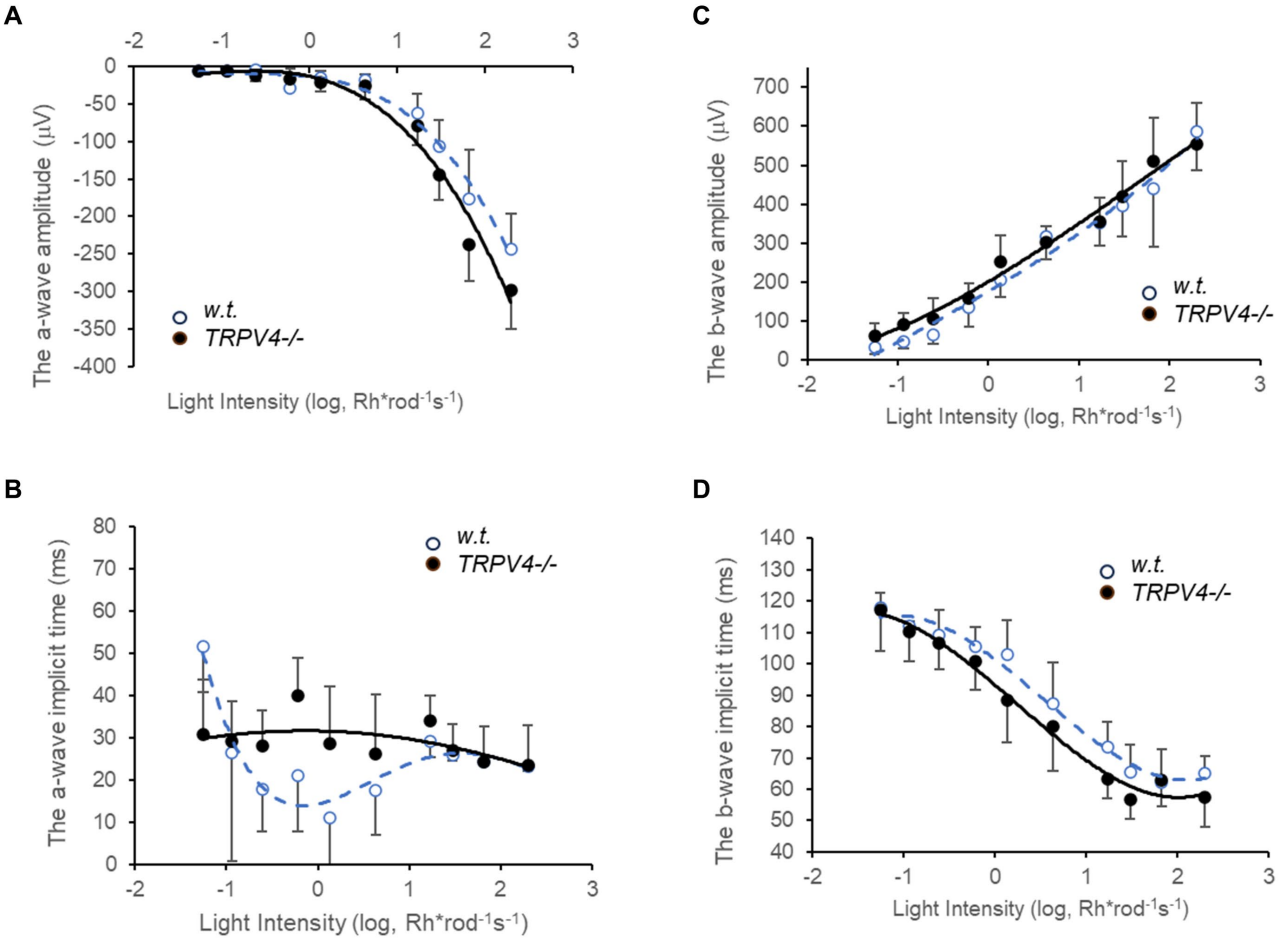
Electroretinography (ERG) evoked by scotopic and mesopic lights in wild-type (*w.t.*) and *TRPV^−/−^*mice dark-adapted overnight. The light intensities are presented as Rh*rod^−1^ s^−1^ in the log unit. **(A,C)** The amplitude of ERG a-wave (**A**, *p* < 0.05) and b-wave (**C**, *p* < 0.05) evoked by scotopic and mesopic light stimuli (0.05 to 200 Rh*rod^−1^ s^−1^) was moderately yet significantly larger in *TRPV4−/−* mice (black dots). **(B,D)** The implicit time was longer for the a-wave (**B**, *p* < 0.01) and shorter for the b-wave (**D**, *p* < 0.01) in the TRPV4 mutants compared to the wild-type mice. The data point at each light intensity was averaged from 8 animals and presented by *mean ± s.d.*, and the data at 10 light intensities were compared between the two species with a two-tail student *t-test* for statistical significance.

The light threshold of mouse rods to the 500 nm light is around 0.22 Rh*rod^−1^ s^−1^ (−6.5 log-unit attenuation of light intensity, log I) (Pang et al., 2010a), the rod photocurrent saturates around 70 Rh*rod^−1^ s^−1^ (−4 log I) (Pang et al., 2010a), and cones are nearly three log unit less sensitive than rods to the light (Yang and Wu, 1996; Pang et al., 2010a). We first applied scotopic to mesopic light flashes (0.05 to 200 Rh*rod^−1^ s^−1^) to record ERG (Figure 2). We compared the data at 10 light intensities (8 pairs of animals at each light intensity) between the wild-type and *TRPV4^−/−^* mice, which revealed an increased amplitude of ERG a- and b-wave (both *p* < 0.05), a longer implicit time for the a-wave (*p* < 0.01), and a shorter implicit time for the b-wave (*p* < 0.01) in *TRPV4^−/−^* mice (Figure 2). At individual light intensities (all *n* = 8 pairs of animals), *TRPV4^−/−^* mice showed a higher amplitude of a-wave at 10^1.4^, 10^1.82^, and 10^2.30^ Rh*rod^−1^ s ^−^1 (*p* = 0.05, 0.015, and 0.049, respectively), a bigger amplitude of b-wave at 10^–0.94^ Rh*rod^−1^ s ^−^1 (*p* = 0.015), a longer implicit time of a-wave at 10^–0.22^ Rh*rod^−1^ s ^−^1 (*p* = 0.004), and a shorter implicit time of b-wave at 10^0.13^ Rh*rod^−1^ s ^−^1 (*p* = 0.047). Given that b-wave is primarily mediated by depolarizing BCs (DBCs) (McCall and Gregg, 2008; Morgans et al., 2009) and OFF responses were not evoked by the brief light stimulation, the data indicates that TRPV4 modulates the scotopic visual signals in rods and RBCs.

**Figure 3:**
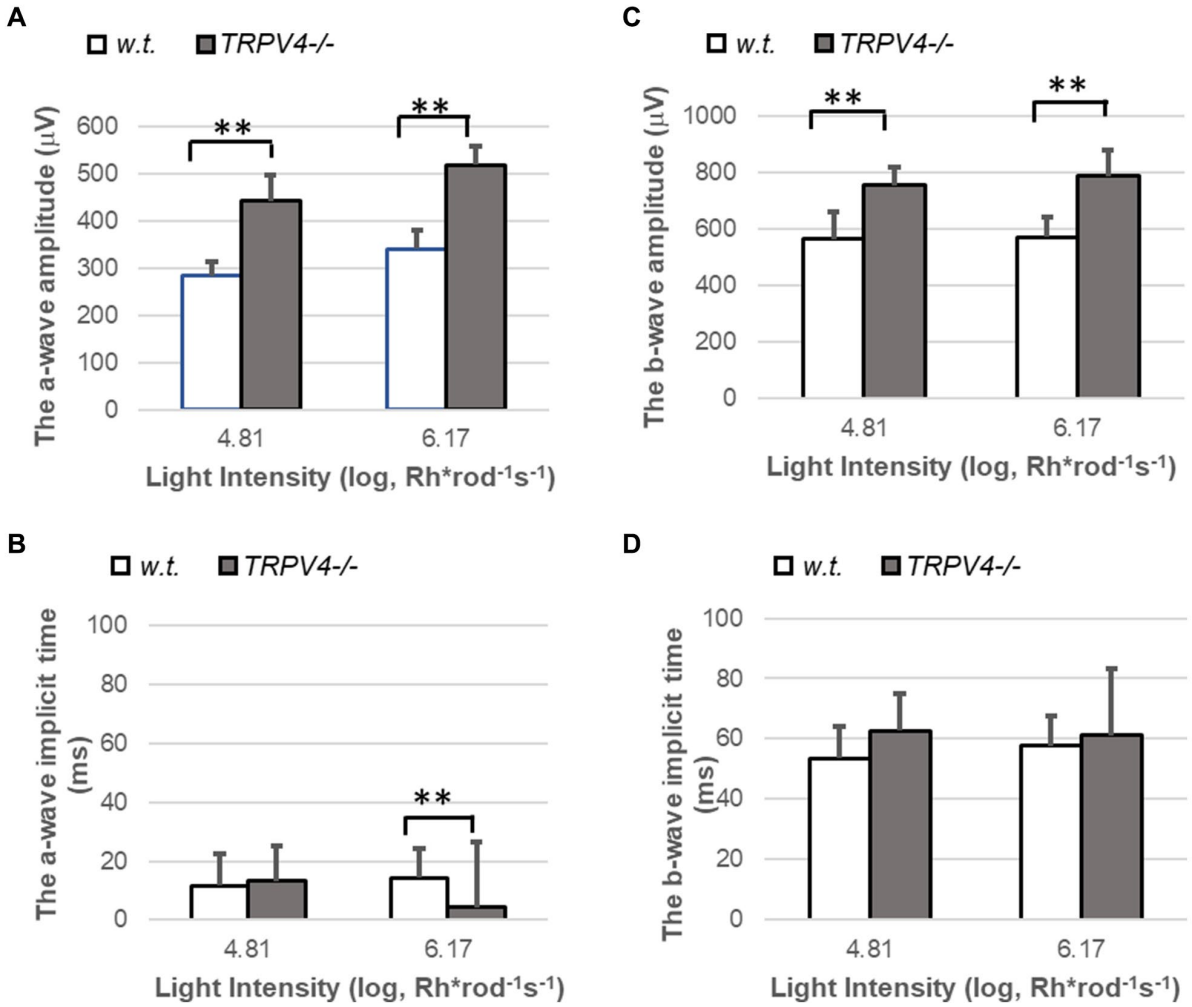
ERG evoked by photopic lights in wild-type (*w.t.*) and *TRPV^−/−^* mice. The light intensities are presented as Rh*rod^−1^ s^−1^ in the log unit. **(A,C)** The amplitude of ERG a-wave (**A**, ^**^p < 0.01) and b-wave (**C**, ^**^p < 0.01) evoked by photopic light stimuli were significantly larger in *TRPV4−/−* mice (gray bars). **(B,D)** The implicit time was shorter for the a-wave evoked by the brightest light (**B**, ^**^p < 0.01) and not changed for the b-wave (**D**, *p* > 0.05) in the TRPV4 mutants compared to the wild-type mice. The bar at each light intensity was averaged from 8 animals and presented by *mean ± s.d.*, and the data at each light intensity was compared between the two species with a two-tail student *t-test* for statistical significance.

We also applied bright white light flashes (10^4.81^ and 10^6.17^ Rh*rod^−1^ s ^−1^) for ERG recording (Figure 3). The amplitude of the a- and b-wave evoked by these photopic lights was significantly larger in *TRPV4^−/−^* mice (both *p* < 0.01, *n* = 8 pairs of animals), but the implicit time of b-wave did not change. The implicit time of a-wave was not altered at the intensity of 10^4.81^ Rh*rod^−1^ s ^−1^ and shorter at the light intensity of 10^6.17^ Rh*rod^−1^ s ^−1^ in *TRPV4^−/−^* mice compared to wild-type mice (*p* < 0.01, *n* = 8 pairs of animals). The data indicates that TRPV4 in wild-type mice reduces the light-evoked hyperpolarization of cones. The effect of TRPV4 on the kinetics of cone signals was different from that on rod signals (Figure 2), which may be associated with the variable synaptic connection of rods and cones.

### TRPV4 regulated the kinetics of rod bipolar cells (RBCs)

RBCs were recorded with the whole-cell patch-clamp techniques from the first soma row in the inner nuclear layer and identified by the long-lasting inward cation currents upon the light of 500 nm, ~0.5 s, and − 4 log I (70 Ph*rod^−1^ s^−1^) and the lack of response to the light offset. The light intensity of −4 log I is near the saturated level for rods and the threshold of M-cones (Pang et al., 2010a).

**Figure 4:**
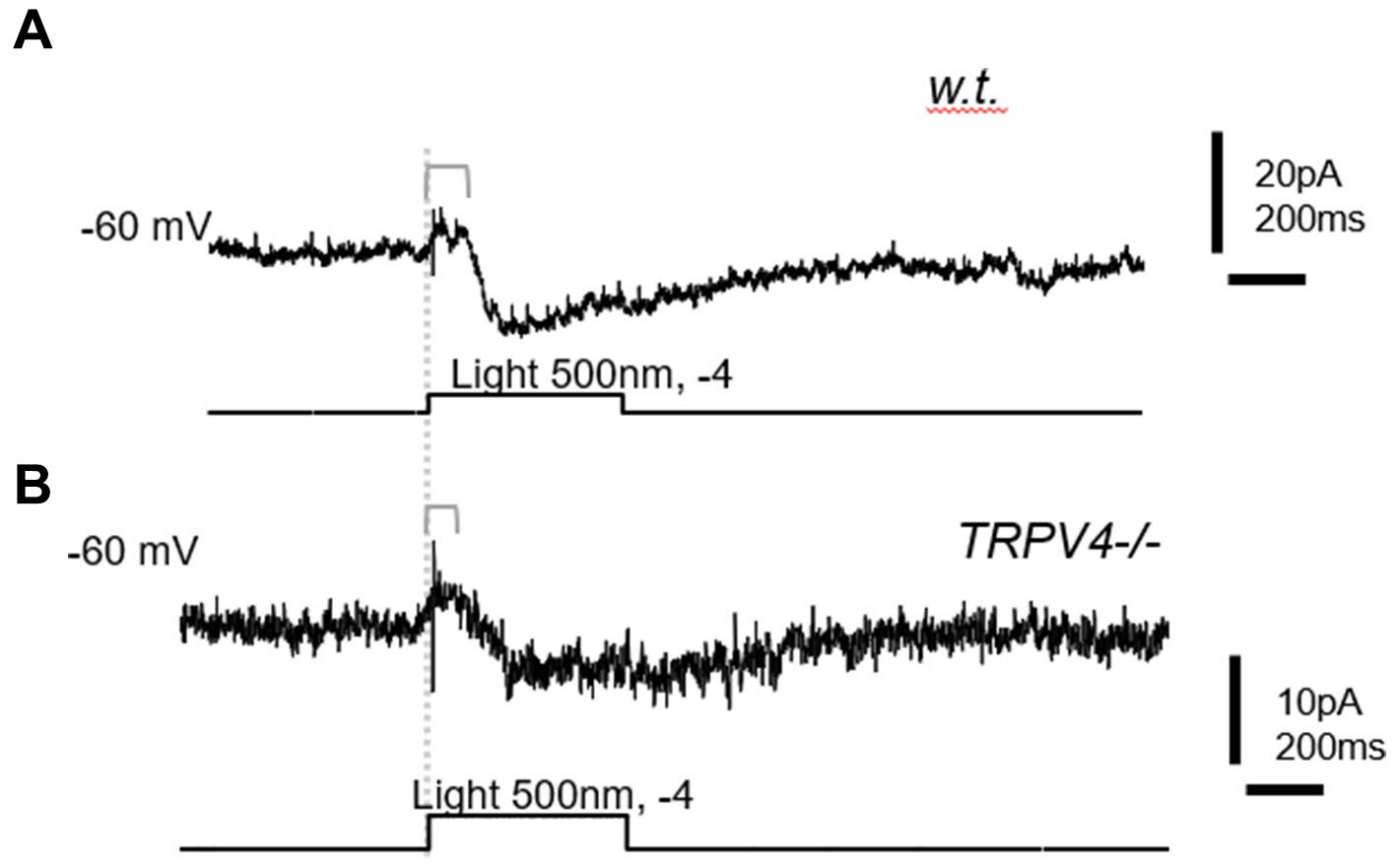
The light-evoked inward cation current (ΔI_C-IN-L_) in rod bipolar cells (RBCs). BCs were recorded with whole-cell patch-clamp techniques. **(A)** Wild-type (w.t.) mice; **(B)** *TRPV4^−/−^* mice. The ΔI_C-IN-L_ was evoked by 500 nm light at the intensity of −4 log I (70 Ph*rod^−1^ s^−1^) and recorded under the voltage clamp mode at the holding potentials of −60 mV. The delay time from the beginning of the light (dashed line) and that of ΔI_C-IN-L_ is longer in the w.t. mouse than in the *TRPV4^−/−^* mouse.

Under voltage-clamp conditions, individual RBCs recorded showed a shorter latency of the light-evoked inward cation current at −60 mV (ΔI_C-IN-L_) in mutant mice (57.13 ± 7.6 ms, *n* = 7), which was significantly shorter than that in wild-type mice (129.1 ± 13.69 ms, *n* = 9) (*p* = 0.001) (Figure 4). This data was consistent with the shorter implicit time of ERG b-wave in the mutant mice. The results indicate that some TRPV4 are active in normal conditions to slow down the kinetics of RBCs.

## Discussion

### Outer retinal neurons express TRPV4

Previous studies have identified TRPV4 in the OPL in mammals (Gilliam and Wensel, 2011; Taylor et al., 2016; Gao et al., 2019; Pang et al., 2021), and TRPV4 protein shows a horizontal distribution pattern in the OPL in the mice, porcine, primate, and salamander retinas (Gilliam and Wensel, 2011; Taylor et al., 2016; Gao et al., 2019; Pang et al., 2021). In the case of acute retinal detachment, the number of apoptotic photoreceptors was reduced by approximately 50% in TRPV4 knockout mice relative to wild-type mice (Matsumoto et al., 2018), which may be attributable to TRPV4 in photoreceptors more than that expressed (Zhao et al., 2015) in retinal pigment epithelium (RPEs). We have observed TRPV4 in photoreceptors and BCs in the salamander retina and BCs in the monkey retina (Gao et al., 2019; Pang et al., 2021). This study, in line with previous findings, revealed TRPV4 in mouse photoreceptors and BCs.

### TRPV4 affects the amplitude and latency of light responses in photoreceptors and BCs

The light threshold of rods to 500 nm light in the mouse retina is around 0.22 Rh*rod^−1^ s^−1^ (−6.5 log I) (Pang et al., 2010a). The rod photocurrent saturates around 70 Rh*rod^−1^ s^−1^ (−4 log I) (Pang et al., 2010a), and cones are nearly three log units less sensitive than rods (Yang and Wu, 1996; Pang et al., 2010a). Given that b-wave is primarily mediated by DBCs (McCall and Gregg, 2008; Morgans et al., 2009) and OFF responses are not evoked by the light flash of ≤5 ms, a- and b-wave evoked by the dim light between 0.05 to 200 Rh*rod^−1^ s^−1^ were primarily mediated by rods and RBCs.

The TRPV4-associated smaller amplitude of a-wave in wild-type mice may be accounted for by the increase in TRPV4-mediated inward current (I_C-IN-TRPV4_) in rods and cones, which could reduce the light-evoked outward current ( $△I_{C}=\;△I_{C-\mathrm{OUT}-L}+I_{C-\mathrm{IN}-\mathrm{TRPV}4}$ ), when light hyperpolarizes the membrane potential (MP) to increase the driving force of TRPV4 ( $△\;E_{\mathrm{TRPV}4}=0-MP$ ). The smaller b-wave in wild-type mice is likely due to the reduced synaptic inputs from photoreceptors to RBCs and other DBCs. TRPV4 showed different effects on the implicit time of a-wave and b-wave in scotopic conditions, and we postulate that such a cell-type specific effect is due to the modulation of TRPV4 on the membrane potential. An inward leakage current via TRPV4 at the background theoretically depolarizes rods to mimic darkness and depolarizes RBCs to mimic light, and this should increase the driving force for ΔI_C-OUT-L_ in rods and reduce the driving force for ΔI_C-IN-L_ in RBCs, shortening the delay in rods and elongating the latency in RBCs. Therefore, TRPV4 expressed in photoreceptors and BCs could explain the effect of TRPV4 on visual signals in outer retinal neurons in scotopic and mesopic conditions.

In a strain of TRPV4 transgenic mice, a previous study (Yarishkin et al., 2018) did not report changes in ERG a- and b-wave evoked by full-field lights of 0.00025–79 cd.s/m^2^, *presumably* white light in the range of 0.03 to 9,559 Rh*rod^−1^ s^−1^ *or* 17.6 to 5.5 × 10^6^ Rh*rod^−1^ s^−1^, and it was uncertain whether the spectrum and intensity of the light stimuli and adaption conditions contributed to the negative results. Rods and RBCs are highly light-sensitive, and light stimuli and background illumination may differentially depolarize cones and rods to variably regulate the driving force of I_C-IN-TRPV4_ in rods and cones. In this study, all our ERG recordings were performed in animals dark-adapted overnight, and our dim light stimuli were 503 nm and focused on testing the rod-driving visual signals.

Horizontal cells (HCs) critically mediate the light adaptation process, and their effect on the implicit time of a- and b-wave evoked by photopic lights is to be ruled out. We have identified TRPV2 protein in HCs and a pressure-evoked inward current in photoreceptors likely mediated by HCs, but HCs have not been reported to express TRPV4. Müller cells express TRPV4 but make no synapses with retinal neurons. Müller cells play a supportive role for retinal neurons, and they can affect neuronal function by regulating glutamate level in extracellular spaces. Our results did not reveal retinal pathologies in mutant retinas at the microscopic level. TRPV4 mediates Na^+^ influxes (Montell, 2005; Jo et al., 2015) to depolarize cells (Fernandez et al., 2013; Ryskamp et al., 2014; Jo et al., 2015; Netti et al., 2017), which in Müller cells could reduce the removal of glutamate by the glutamate transporter GLAST (Bringmann et al., 2006) as the transportation relies on the energy stored in the Na^+^ electrochemical gradient (Akyuz et al., 2015). On the other hand, BCs make invaginating synapses with rods and both invaginating and flat synapses with cones (Dowling, 2012; Behrens et al., 2016; Xiao et al., 2023), and the glutamate released from rods in scotopic conditions is relatively low and better restricted to the ribbon synapses due to the synaptic structure. Thus, we propose that TRPV4 in Müller cells may contribute to the effect of TRPV4 on the photopic b-wave but is less accountable for the changes in photopic a-waves and the a- and b-waves elicited by dim lights.

Suppressing TRPV4 did not reduce the light response of outer retinal neurons at the populational level up to 7 months. Whether it enhances the mechanical vulnerability of outer retinal neurons or causes compensating expression of other MSCs is to be further explored.

### The effect of TRPV4 is dynamic and cell-type specific

TRPV4 may desensitize, but TRPV4 opens at temperatures above ∼27°C. When constantly exposed to 37°C, TRPV4 could still respond to increased temperatures, showing incomplete desensitization. Thus, TRPV4 was thought to be constitutively active at body temperature (Guler et al., 2002; Nilius et al., 2004). Our results revealed the effect of TRPV4 on the normal light response of photoreceptors and BCs, which, consistent with the previous findings, indicates that some TRPV4 channels are constitutively active in physiological conditions.

Based on our results and a reversal potential ~0 mV and certain open probability of TRPV4 (Strotmann et al., 2000; Suzuki et al., 2003b; Nilius et al., 2004; O'Neil and Heller, 2005; Cao et al., 2009; Gao et al., 2019; Kashio and Tominaga, 2022), we propose a novel functional mechanism for TRPV4: TRPV4 depolarizes the membrane to regulate the dark membrane potential of photoreceptors and DBCs and the implicit time of light responses, and the light-induced hyperpolarization in photoreceptors enhances the driving force of TRPV4, which could decrease the amplitude of the light response of photoreceptors, RBCs, and DBCs.

In addition, in around 10% of TRPV4 mutant mice, we observed one undeveloped eye. The pathological mechanism is unclear. The animal facilities in our institute are fully credited and have expertise and experience in housing mice and other animals. Factors like nutrition, environment, and injury can affect the development of the eye. On the other hand, these factors are less likely to cause unilateral and complete missing of an eyeball. Lowe syndrome (OCRL, MedGen UID: 18145; Concept ID: C0028860) is a rare X-linked congenital disease that presents congenital cataracts and glaucoma. One or both eyeballs are abnormally small (microphthalmia), and in some affected individuals, the eyeball may appear to be completely missing. OCRL is an inositol polyphosphate 5-phosphatase, which is mutated in Lowe syndrome. Studies on Lowe syndrome have suggested that OCRL may act through regulation of TRPV4 (Luo et al., 2014; Jing et al., 2024), and a novel disease-causing OCRL allele prevents TRPV4-mediated calcium signaling. Our data are generally in line with these data from patients and animal models.

## Data availability statement

The original contributions presented in the study are included in the article/supplementary material, further inquiries can be directed to the corresponding author.

## Ethics statement

The animal study was approved by Institutional Animal Care and Use Committee. The study was conducted in accordance with the local legislation and institutional requirements.

## Author contributions

YL: Formal analysis, Investigation, Writing – original draft. MK: Formal analysis, Investigation, Writing – original draft. SW: Resources, Writing – review & editing. J-JP: Conceptualization, Funding acquisition, Investigation, Methodology, Project administration, Resources, Supervision, Validation, Writing – original draft, Writing – review & editing.
